# Influence of Personal, Environmental, and Community Factors on Cigarette Smoking in Adolescents: A Population-Based Study from Taiwan

**DOI:** 10.3390/healthcare10030534

**Published:** 2022-03-14

**Authors:** Yu-Chun Liang, Jung-Yu Liao, Charles Tzu-Chi Lee, Chin-Mei Liu

**Affiliations:** 1 Department of Health Promotion and Health Education, National Taiwan Normal University, Taipei 10610, Taiwan; monica.liang@healthconn.com (Y.-C.L.); lee@ntnu.edu.tw (C.T.-C.L.); 2 Department of Public Health, Kaohsiung Medical University, Kaohsiung City 80708, Taiwan; jyliao@kmu.edu.tw; 3 Taiwan Centers for Disease Control, Taipei City 10050, Taiwan

**Keywords:** adolescent, cigarette smoking, second hand smoking (SHS), logistic regression, path analysis

## Abstract

Understanding the factors that influence cigarette smoking among adolescents is critical. We identified personal, community, and environmental factors associated with current cigarette smoking among adolescents. This population-based cross-sectional analysis study was conducted using the 2012 Taiwan Global Youth Tobacco Survey and the sociodemographic statistics of the city or county from Taiwan’s Ministry of the Interior. A total of 27,524 participants (age: 12–18-years) was included. The associated factors were identified through logistic regression. A path analysis was performed to examine the pathway from the associated factors to current cigarette smoking. According to this analysis, the following factors were prominently and positively associated with adolescent cigarette smoking: one personal factor (pocket money), five environmental factors (home secondhand smoke (SHS) exposure, smoker friends, outside SHS exposure, school SHS exposure, and smoker parents), and two community factors (free cigarettes from tobacco companies and indigenous population). By contrast, five personal factors (feeling less comfortable smoking at social occasions, feeling indifferent about smoking or not smoking at social occasions, female sex, feeling that quitting is difficult, and feeling that quitting after having smoked is harmful to health) and one environmental factor (school antismoking education) had negative effects. Thus, comprehensive interventions promoting the perception of harm caused by smoking and interrupting access to cigarettes through social networks can reduce cigarette smoking in adolescents.

## 1. Introduction

Since the recognition of cigarette smoking as the leading cause of preventable disease and death [1], its prevalence has progressively declined in Taiwanese adolescents [2]. The implementation of the Anti-Smoking Act may have limited results for modern teens due to the increased availability of alternative tobacco products (such as e-cigarettes) and advertising targeted at teenagers [3]. Nearly 80% people who have ever smoked daily smoked their first cigarette before they were 18 years old [4,5] and more than one-third of the adults who have ever smoked a cigarette smoked their first cigarette were 14 years of age [6]. Earlier smoking initiation was associated with sustained smoking through adulthood [6]. Thus, understanding the factors that influence adolescent cigarette smoking in adolescents is crucial. Research has highlighted the importance of multilevel influential factors for determining health behaviors in adolescents [7,8]. According to the socioecological developmental model [9] and social cognitive theory [10], smoking behavior is a result of interactions among multilevel factors (i.e., personal, community, and environmental) [8,11,12]. Focusing on these multilevel factors is critical to the success of antismoking efforts in adolescents.

The personal factors associated with and demographic differences in cigarette smoking among adolescents have been explored. The smoking rate is higher among men than among women [11,13,14], and it is higher among older adolescents than among younger adolescents [11,15]. Weekly pocket money may facilitate smoking initiation in adolescents [16]. Knowledge of smoking and attitudes toward smoking are negatively related in adolescents. A study revealed that adolescents with a more negative attitude toward smoking are less likely to be current smokers [17]. Moreover, in another study, people with poor knowledge of and positive attitudes toward smoking were predicted to increase cigarette smoking in the subsequent 5 years [18]. However, in Botswana, increased awareness regarding the harm caused by smoking had no protective effect on cigarette smoking behavior in adolescents [19].

Environmental factors, such as exposure to secondhand smoke (SHS), exposure to antismoking information, and parents’ and peers’ smoking status, may influence adolescent cigarette smoking. Reducing SHS exposure has helped adolescents increase their antismoking convictions, improve their smoking behaviors, and increase their willingness to cease smoking [20]. Local and household smoke-free policies can also significantly protect adolescents from becoming established smokers [21,22]. Moreover, antismoking information provides knowledge about smoking risks and influences attitudes toward cigarette smoking [23], which may reduce the likelihood of smoking initiation among adolescents [24]. The smoking behaviors of parents and peers are significantly related to cigarette smoking in adolescents. Parents or friends who smoke tend to influence adolescents’ initial smoking behavior over the developmental periods from junior high school to high school [23,25]. In a study that investigated the reasons for the transition from e-cigarette use to cigarette smoking among nonsmoking young adults, sharing cigarettes with and accessing cigarettes from peers were among the main reasons [26]. Taken together, family and peers are the key influential factors in the initiation or maintenance of cigarette use.

The community factors that influence adolescent cigarette smoking also warrant consideration. Tobacco advertising is one such community factor; it increases adolescents’ awareness of opportunities to access cigarettes [6,19]. Moreover, tobacco advertising and promotion create an impression among youths that the products are appropriate and that smoking, especially among peers, is stylish, fashionable, and acceptable [27]. Exposure to indirect tobacco advertising, promotion, and sponsorship (TAPS) is also strongly associated with smoking initiation in adolescents [28]. An Austrian study [29] reported that the prevalence of smoking in the indigenous population was more than double that in the nonindigenous population because of social pressure to smoke and the social exclusion associated with quitting. Therefore, adolescents from areas with a high indigenous population may be more exposed to smoking. Moreover, in numerous workplaces, smoking is part of the organizational culture.

The aforementioned multilevel factors have been found to influence cigarette smoking in adolescents. However, to the best of our knowledge, the associations between these factors and cigarette smoking behavior have rarely been studied through path analysis. This study identifies personal, community, and environmental factors associated with and influencing current cigarette smoking among adolescents by constructing relevant pathways.

## 2. Materials and Methods

### 2.1. Study Design and Population

#### 2.1.1. Data Source

The Taiwan Global Youth Tobacco Survey (GYTS) is a national sample of Taiwanese middle- and high-school students (i.e., 12–18-year-old junior high, senior high, vocational school, and night-school students) from all 22 cities and counties of Taiwan. This GYTS was developed by the Taiwan Health Promotion Administration in conjunction with the US Center for Disease Control and Prevention for surveillance of tobacco use behavior, perceptions, and attitudes. It involves a self-administered questionnaire with items focusing on cigarette smoking behavior, perceptions, and attitudes toward smoking hazards, SHS exposure, and smoking ban implementation on campuses. Basic demographic variables are also recorded. In 2012, the Taiwan GYTS used a multistage sample design using regional-level stratification, with schools selected in proportion to their enrolment sizes and a representative sample of students was also selected. The applicable classrooms were randomly chosen from within the selected schools, and all students in these selected classrooms were eligible for participation. Moreover, sociodemographic statistics on each city or county in which the schools were located were collected from Taiwan’s Ministry of the Interior.

#### 2.1.2. Study Design

This was a population-based cross-sectional analysis study.

#### 2.1.3. Study Population

The questionnaire from the 2012 Taiwan GYTS was completed by 34,552 students, with an overall response rate of >90.0%. Participants aged >18 years and with missing data were excluded. Thereafter, responses of only 27,524 participants were included.

### 2.2. Measures

#### 2.2.1. Outcome

Current cigarette smoking was defined through self-report of having smoked cigarettes on at least 1 day in the past 30 days. The related question in the questionnaire was “How many days in the past 30 days did you smoke?” The participants responding to this question with 1 or more days were categorized as current cigarette smokers.

#### 2.2.2. Potential Associated Factors

The following potential associated factors were evaluated: personal (knowledge of health risks related to smoking and harmful effects of SHS and attitudes toward smoking); environmental (parents and peers who smoke, exposure to school antismoking education, quantity of SHS exposure (days of SHS exposure at home, school, and outside of home and school over the past 7 days)); and community (TAPS exposure, sociodemographic statistics of the school region (i.e., indigenous population, low-income population, college and university graduate population, migrant population, temple density, crime rates, fertility rate of under 19 years old, divorce rate, and communicable diseases burden)).

### 2.3. Statistical Analysis

The chi-square test was used to compare the distributions of personal, community, and environmental factors among current and noncurrent smoking participants. Logistic regression analysis was used to explore the adjusted effect of explanatory variables on current smoking after mutual controlling. Odds ratios (ORs) and their 95% confidence intervals (CIs) are reported. Values of *p* < 0.05 were considered statistically significant.

After the predicting factors were ascertained through logistic regression, path analysis was performed to examine the pathways from the possible factors to current cigarette smoking. Modification indices were used to assess the model fit. Five statistical tests were used to evaluate the overall goodness-of-fit of the correction model: standardized root mean square residual, root mean square error of approximation, comparative fit index, Tucker–Lewis index, and parsimonious goodness-fit-index. After the model was corrected, the effects of factors associated with adolescents’ current smoking were estimated and significantly positive and negative associations were reported. Finally, all data were split into two data sets (training and testing) to evaluate the consistency of the results through validation analysis. The data analysis was performed using SPSS (version 23) and Amos (version 21).

### 2.4. Ethical Approval

The Institutional Review Board of National Taiwan Normal University (Protocol Number: 201902HM005) approved the study. Written consent was exempted because the data were obtained from Taiwan Youth Tobacco Survey, which contains de-identified secondary data released for research purposes.

## 3. Results

### 3.1. Sample Characteristics

Table 1 presents the results of the descriptive analysis of the background information and study variables. Overall, 27,524 students were included in the analysis. Of these, 50% were females, and 49.1% were aged 13–15 years. Approximately 9.2% of the participants were current smokers, of which 7.0% were aged 13–15 years and 11.3% were 16–18 years. Of the current smokers, most (80.5%) had received antismoking education at school; of these, 74.9% were considering ceasing smoking because of its harmful effects and 94.8% reported knowing that SHS is harmful. Furthermore, the parents of 65.7% of the current smokers were smokers themselves, and most of the friends of the 39.4% of the current smokers were also smokers. Moreover, 52.6% and 62.3% of the participants reported SHS exposure at school and home, respectively, over the previous 7 days, and 23.8 % had received free cigarettes from a tobacco company. The distribution of these factors was significantly different between the nonsmokers and smokers.

### 3.2. Factors Associated with Current Cigarette Smoking

Table 2 presents the results of the multivariate logistic regression analysis of the personal, community, and environmental factors in terms of background. Most of the associated factors were somewhat linked to current cigarette smoking. The odds of current cigarette smoking were significantly positively related to personal factors, including pocket money (>NT$4500: OR 4.21, 95% CI 3.43–5.18, *p* < 0.001). The following environmental factors were significantly related to the likelihood of cigarette smoking: both parents were smokers (OR 1.65, 95% CI 1.38–1.97, *p* < 0.001), most friends smoked (OR 36.27, 95% CI 27.25–48.29, *p* < 0.001), >5 days of school SHS exposure (OR 2.37, 95% CI 2.06–2.71, *p* < 0.001), >5 days of home SHS exposure (OR 1.19, 95% CI 1.03–1.37, *p* = 0.016), and >5 days of SHS exposure outside of the home and school (OR 3.36, 95% CI 2.82–4.00, *p* < 0.001). The community factor indigenous population was associated with an increased likelihood of current smoking (OR 1.01, 95% CI 1.01–1.02, *p* < 0.001). Moreover, adolescents from areas where tobacco companies offered free cigarettes presented significantly higher smoking risk than those from other areas (*p* < 0.001). Current cigarette smoking was lower for some personal factors, such as female gender (OR 0.54, 95% CI 0.48–0.60, *p* < 0.001) and low acceptability of smoking during social occasions (OR 0.31, 95% CI 0.26–0.35, *p* < 0.001). School antismoking education and rules, which are community factors, were associated with a lower likelihood of current smoking (OR 0.74, 95% CI 0.62–0.89, *p* = 0.001).

### 3.3. Effects of Associated Factors on Current Cigarette Smoking

**Figure 1:**
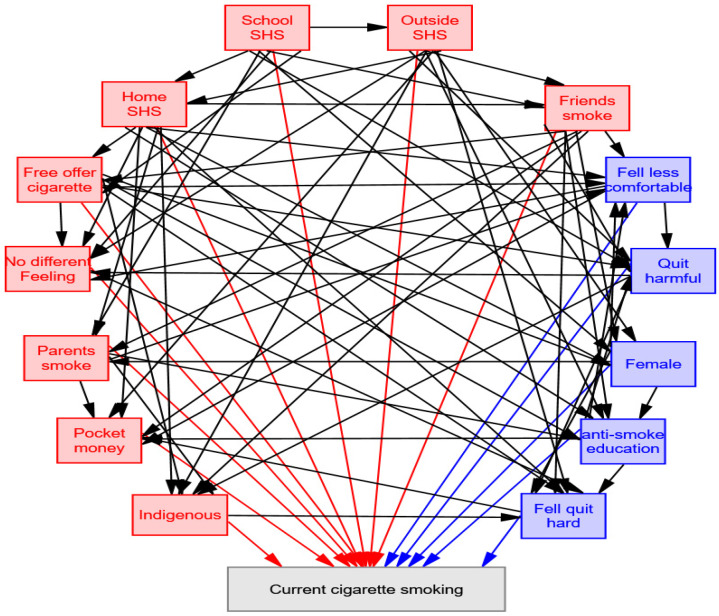
Final path analysis model for factors associated with current cigarette smoking.

According to the goodness-of-fit statistics, the final correction models were acceptable (Supplementary Table S1). Figure 1 presents the final path analysis model of the total effects on current cigarette smoking incidence. Table 3 presents the factors associated with direct, indirect, and total effects on current cigarette smoking incidence. Eight associated factors exhibited a direct positive effect on smoking incidence: free cigarettes from tobacco companies (0.182), home SHS exposure in the previous 7 days (0.153), smoker friends (0.104), SHS exposure outside of the home and school in the previous 7 days (0.061), school SHS exposure in the previous 7 days (0.046), indigenous population (0.037), smoker parents (0.019), and pocket money (0.08). Six associated factors displayed a direct negative effect on smoking incidence: feeling less comfortable smoking on social occasions (−0.236), feeling indifferent to smoking or not smoking on social occasions (−0.170), female gender (−0.068), feeling that quitting is difficult (−0.047), receipt of school antismoking education (−0.021), and feeling that quitting after having smoked in harmful to health (−0.015). The training model results were consistent with the results of the testing model (Supplementary Table S2) and with original data.

## 4. Discussion

In this study, we examined the personal, environmental, and community factors associated with current cigarette smoking in adolescents through multivariate logistic regression and path analyses. After simultaneously assessing the associations, one personal factor (pocket money), five environmental factors (home SHS exposure, smoker friends, SHS exposure, school SHS exposure outside of the home and school, and smoker parents), and two community factors (free cigarettes from tobacco companies and indigenous population) were found to be associated with a significant increase in cigarette smoking incidence. By contrast, five personal factors (feeling less comfortable smoking on social occasions, feeling indifferent about smoking or not smoking on social occasions, female gender, feeling that quitting is difficult, and feeling that quitting after having smoked is harmful to health), and one environmental factor (antismoking education) negatively affected current smoking behavior. The factors affecting youth smoking during the developmental stages of adolescence are highly complicated. Reducing smoking among adolescents may require comprehensive interventions tailored toward the promotion of adolescent perceptions regarding the harmful effects of smoking, as well as toward preventing tobacco companies from advertising through social media or offering free cigarettes to adolescents.

Cigarette smoking is linked to gender. The present study confirmed a previous finding that the rates of current cigarette smoking are lower in females [30] However, this result is inconsistent with the worldwide trend, in which no gender difference has been noted in adolescents’ smoking behavior. In the sociocultural context, Taiwan has an East Asian culture, in which the social acceptance of smoking by women is low. By contrast, in various Western countries, smoking among women is as acceptable as it is among men. We also observed that teenagers who felt less comfortable smoking during social occasions had a lower likelihood of being current smokers. However, this observation may represent the sociocultural influence of smoking behavior on teenagers, as a result of which they are reluctant to publicly reveal their smoking habit.

Smoking is a learned behavior. Better knowledge of and attitudes toward smoking have positive effects on current smoking in adolescents. Increased access to antitobacco information at school can increase students’ knowledge of its risks and change their attitudes toward smoking [31]. Lower levels of knowledge on smoking hazards [32] and positive attitudes toward smoking [33] promote smoking among Asian adolescents. A GYTS in South Asia suggested that adolescents who are not informed about the harmful effects of cigarette smoking at school or home are more likely to smoke [34]. We observed that nonsmokers who received school antismoking education believed that quitting was difficult, and believed that it was harmful to smoke and then quit. Thus, establishing antismoking behaviors through education is valuable. Antismoking intervention programs have led to a decrease in smoking behaviors [35,36]. Such programs provide smoking-related health information through mass media, schools. In general, success with a single strategy is difficult. For instance, a study investigating education about the harm caused by smoking in schools reported no association of this education with current smoking in adolescents [37]. The results might have been inconsistent due to other factors, including SHS exposure or the desire to mimic adult behavior, despite knowledge of the risks [38].

Parents and peers can influence smoking in adolescents. Having smoker parents and peers was positively associated with smoking initiation in adolescents [39] and higher smoking susceptibility; this is also a significant predictor of adolescents’ current smoking status [40]. The risk of smoking in childhood and adolescence increases if at least one parent smokes, and this risk increases almost threefold when both parents are smokers [41]. Social development can also influence adolescents’ smoking behavior. Students who are under peer pressure are more likely to smoke cigarettes [42]. A 3-year cohort study investigated the effect of parent and peer influence on adolescent smoking and found no parental effect with age. This study also indicated that best friends and friends had a stronger influence on younger adolescents, whereas friends and same-grade students had a stronger influence on older adolescents [43]. Moreover, smoker parents and friends were strong determinants of all types of SHS exposure [44]. SHS exposure is a significant risk factor for current smoking in adolescents, and increases the risk of cigarette smoking among young adolescents [45]. Under the Tobacco Harm Prevention Act, smoking is completely prohibited in most indoor or outdoor public spaces in Taiwan, except in designated smoking areas. However, some private spaces that are free of law enforcement, such as homes, could be major areas of SHS exposure for youths if their parents smoke. Some SHS exposure among youths could be unintentional. If someone lives in a smoking family, their parents are less likely to ban smoking and may even provide adolescents with tobacco products. In such cases, adolescents are in a vulnerable position, and incapable of avoiding such smoking environments. A shown in Table 3, SHS and contact with smokers influence youth smoking. Home SHS exposure implies frequent exposure to smokers. Our findings also suggest that SHS exposure is associated with an increased likelihood of current smoking in adolescents. Comprehensive smoke-free legislation that prohibits smoking in all indoor public spaces, including schools, workplaces, bars, and restaurants, can reduce SHS exposure and encourage people to quit smoking [46].

Easy access to cigarettes is a key factor contributing to smoking initiation among adolescents [47]. Access to free cigarettes, either from tobacco companies or smoker friends, was the strongest predictor of cigarette smoking in this study. Selling or providing tobacco products to younger people is illegal in Taiwan. Nonetheless, adolescents, particularly current smokers, reported that they were able to obtain cigarettes from retail outlets, such as convenience stores, supermarkets, tobacco sales, cyber cafés, weddings, funerals, Kondatam television (KTV), and music television, in the 30 days prior to the questionnaire, and approximately 3–5% of smokers had been offered free cigarettes, which implies that strict regulation is necessary. This is valuable information for policy makers because it highlights the need for surveillance in the retail industry. Adolescents’ exposure to TAPS is also a critical factor influencing smoking initiation. The promotional activities of tobacco companies, particularly their distribution of free cigarettes, were significantly associated with increased smoking risk [48]. Adopting and enforcing interventions that prevent adolescents from accessing tobacco products should be included in strategies for reducing smoking initiation. Adolescents are more sensitive to cigarette pricing policies [49]. Pocket money was positively associated with current smoking. A cigarette pricing policy could bar adolescents from accessing cigarettes [50].

The path analysis in this study was not used to test the overall model validity but to clarify the specific direct and indirect relationships across the variables. Our study provides a possible relational structure but does not eliminate other possible modes. Despite its limitations, this study has several key strengths. We used a two-step analysis to examine the pathway from associated factors to current cigarette smoking; this has rarely been reported. After the consideration of all the possible associations, our findings on the associations of personal, environmental, and community factors with cigarette smoking in adolescents were consistent with prior results. These factors, particularly access to free cigarettes, smoker friends, and SHS exposure, play a major role in tobacco use among adolescents. More comprehensive antismoking acts and effective strategies are required to prevent adolescent cigarette smoking.

In conclusion, the present study demonstrated the influences of personal, environmental, and community factors on current smoking among Taiwanese students aged 12–18 years, and found that single interventions were not effective. Comprehensive interventions promoting the perception of the harmful effects of smoking, as well as interrupting access to cigarettes through social networks, can reduce cigarette smoking in adolescents. Taiwan follows the framework convention on tobacco control implemented by the Tobacco Harm Prevention Act, promoting various preventive measures. Therefore, the prevalence of smoking among 12–15-year-olds and 16–18-year-old adolescents decreased to 61% and 43%, respectively, from 2008 to 2019. However, the perception of cigarette smoking as being trendy and cool and the access to advanced-technology electronic products, such as electronic cigarettes, are influencing young people to use cigarettes. SHS exposure, smokers, and the offer of free cigarettes to adolescents combine to influence youth smoking, and comprehensive control is urgently required.

## Figures and Tables

**Table 1:** Characteristics of non-smokers and smokers (*n* = 27,524).

Characteristic	Non-Smoker	Smoker	*p* Value
	*n* = 24,995	*n* = 2529	
**Personal factors**			
Gender, *n* (%)			
Male	11,896 (47.6)	1816 (71.8)	<0.001
Female	13,099(52.4)	713 (28.2)	
Age, *n* (%)			
12–15 years old	12,571 (50.3)	941 (37.2)	<0.001
16–18 years old	12,424 (49.7)	1588 (62.8)	
Pocket money, *n* (%)			
none	6065 (24.3)	194 (7.7)	<0.001
less than 500 NTD ^1^	7142 (28.6)	445 (17.6)	
500~1499 NTD ^1^	5714 (22.8)	491 (19.4)	
1500~2499 NTD ^1^	2647 (10.6)	363 (14.4)	
2500~3499 NTD ^1^	1450 (5.8)	287 (11.3)	
3500~4499 NTD ^1^	758 (3.0)	173 (6.8)	
more than 4500 NTD ^1^	1219 (4.9)	576 (22.8)	
Feel quitting hard, *n* (%)			
No	7180 (28.7)	1054 (41.7)	<0.001
Yes	17815 (71.3)	1475 (58.3)	
Feel comfortable when smoking at social occasions, *n* (%)			
More comfortable	1940 (7.8)	638 (25.2)	<0.001
Less comfortable	12954 (51.8)	580 (22.9)	
No difference, whether smoking or not	10101 (40.4)	1311 (51.9)	
Smoke then quit harmful, *n* (%)			
No	4517 (18.1)	635 (25.1)	<0.001
Yes	20,478 (81.9)	1894 (74.9)	
Feel SHS harmful, *n* (%)			
No	754 (3.0)	131 (5.2)	<0.001
Yes	24,241 (97.0)	2398 (94.8)	
**Environmental factors**			
Parents’ smoking status, *n* (%)			
None	13,411 (53.7)	867 (34.3)	<0.001
Both	1736 (6.9)	465 18.4)	
Only father	9464 (37.9)	1122 (44.3)	
Only mother	384 (1.5)	75 (73.0)	
Friends’ smoking status, *n* (%)			
None	10,808 (43.2)	57 (2.2)	<0.001
Some	13,021 (52.1)	1375 (54.4)	
Most	1077 (4.3)	997 (39.4)	
All	89 (0.4)	100 (4.0)	
School anti-smoking education, *n* (%)			
None	3456 (13.8)	493 (19.5)	<0.001
This semester	4769 (19.1)	590 (23.3)	
The past semester	8168 (32.7)	757 (29.9)	
Two semesters ago	4503 (18.0)	313 (12.4)	
Three semesters ago	1449 (5.8)	118 (4.7)	
Two years or longer ago	2650 (10.6)	258 (10.2)	
School SHS past 7 days, *n* (%)			<0.001
None	20,890 (83.6)	1199 (47.4)	
1–2 days	2124 (8.5)	325 (12.9)	
3–4 days	730 (2.9)	225 (8.9)	
>5 days	1251 (5.0)	780 (30.8)	
Home SHS past 7 days, *n* (%)			<0.001
None	15,492 (62.0)	953 (37.7)	
1–2 days	2412 (9.6)	224 (8.9)	
3–4 days	1526 (6.1)	138 (5.4)	
>5 days	5565 (22.3)	1214 (48.0)	
Outside of home and school SHS past 7 days, *n* (%)			<0.001
None	8940 (35.8)	202 (8.0)	
1–2 days	5761 (23.0)	259 (10.2)	
3–4 days	3275 (13.1)	300 (11.9)	
>5 days	7019 (28.1)	1768 (69.9)	
**Community factors**			
where to receive free cigarettes offered by tobacco companies, *n* (%)			
Never obtained	24,089 (96.4)	1927 (76.2)	<0.001
Convenience store, supermarket, department store	114 (0.5)	96 (3.8)	
Tobacco sale	54 (0.2)	38 (1.5)	
Internet café	108 (0.4)	91 (3.6)	
KTV or MTV	46 (0.2)	69 (2.7)	
On the side of the road	79 (0.3)	42 (1.7)	
Wedding or funerals	338 (1.3)	140 (5.5)	
Other places	167 (0.7)	126 (5.0)	
Indigenous population, mean (95% CI)	3.6 (3.6–3.8)	5.7 (5.3–6.1)	<0.001
Low-income population, mean (95% CI)	1.6 (1.5–1.6)	1.8 (1.7–1.8)	<0.001
College/university, graduate population, mean (95% CI)	85.5 (85.4–85.5)	85.5 (85.4–8515)	0.47
Migrant population, mean (95% CI)	3.9 (3.9–4.0)	3.9 (3.9–4.0)	0.83
Temple density, mean (95% CI)	0.7 (0.6–0.7)	0.6 (0.6–0.7)	<0.001
Criminal cases, mean (95% CI)	3.8 (3.7–3.8)	3.8 (3.7–3.8)	0.47
Fertility rate under 19 years old, mean (95% CI)	4.6 (4.4–4.5)	5.0 (4.9–5.0)	<0.001
Divorce rate, mean (95% CI)	2.3 (2.2–2.3)	2.3 (2.3–2.3)	0.003
Communicable diseases, mean (95% CI)	4.2 (4.1–4.2)	4.2 (4.2–4.3)	0.19

^1^ US$1 = NT$31.1 in 2019. SHS = secondhand smoke. CI = confidence interval.

**Table 2:** Factors associated with current cigarette smoking in adolescents (*n* = 2529).

Variables	Unadjusted Analysis	*p* Value	Adjusted Analysis	*p* Value
	Odds Ratio (95% CI ^a^)		Odds Ratio (95% CI ^a^)	
**Personal factors**				
Gender (female vs. male)	0.36 (0.33–0.39)	<0.001	0.54 (0.48–0.60)	<0.001
Age (16–18 years old vs. 13–15 years old)	1.71 (1.57–1.86)	<0.001		
Pocket money				
Less than 500 NTD (vs. none)	1.95 (1.64–2.31)	<0.001	2.12 (1.74–2.57)	<0.001
500–1499 NTD (vs. none)	2.69 (2.27–3.18)	<0.001	2.30 (1.89–2.79)	<0.001
1500–2499 NTD (vs. none)	4.29 (3.58–5.13)	<0.001	2.94 (2.39–3.62)	<0.001
2500–3499 NTD (vs. none)	6.19 (5.11–7.50)	<0.001	3.01 (2.41–3.76)	<0.001
3500–4499 NTD (vs. none)	7.14 (5.74–8.88)	<0.001	3.26 (2.52–4.22)	<0.001
More than 4500 NTD (vs. none)	14.77 (12.41–17.58)	<0.001	4.21 (3.43–5.18)	<0.001
Feel quitting hard (yes vs. no)	0.56 (0.52–0.61)	<0.001	0.65 (0.58–0.72)	<0.001
Feel comfortable at social occasions				
Less comfortable (vs. more comfortable)	0.14 (0.12–0.15)	<0.001	0.31 (0.26–0.35)	<0.001
no difference (vs. more comfortable)	0.40 (0.36–0.44)	<0.001	0.51 (0.45–0.58)	<0.001
Smoked then quit harmful (yes vs. no)	0.66 (0.60–0.72)	<0.001	0.82 (0.73–0.93)	0.001
Feel SHS is harmful (yes vs. no)	0.57 (0.47–0.69)	<0.001		
**Environmental factors**				
Parents’ smoking status				
Both (vs. none)	4.14 (3.66–4.69)	<0.001	1.65 (1.38–1.97)	<0.001
Only father (vs. none)	1.83 (1.67–2.01)	<0.001	1.12 (0.98–1.28)	0.088
Only mother (vs. none)	3.02 (2.34–3.91)	<0.001	1.48 (1.07–2.05)	0.019
Friends’ smoking status				
Some (vs. none)	20.02 (15.34–26.12)	<0.001	8.54 (6.51–11.21)	<0.001
Most (vs. none)	175.52 (133.43–230.89)	<0.001	36.27 (27.25–48.29)	<0.001
All (vs. none)	213.04 (144.76–313.54)	<0.001	30.12 (19.04–47.63)	<0.001
School anti-smoking education and rules				
This semester (vs. none)	0.87 (0.76–0.99)	0.028	0.92 (0.79–1.08)	0.335
The past semester (vs. none)	0.65 (0.58–0.73)	<0.001	0.82 (0.70–0.95)	0.009
Two semesters ago (vs. none)	0.49 (0.42–0.57)	<0.001	0.74 (0.62–0.89)	0.001
Three semesters ago (vs. none)	0.57 (0.46–0.70)	<0.001	0.80 (0.62–1.03)	0.087
Two years or longer ago (vs. none)	0.68 (0.58–0.80)	<0.001	0.81 (0.67–0.99)	0.038
School SHS during the past 7 days				
1–2 days (vs. none)	2.67 (2.34–3.04)	<0.001	1.42 (1.22–1.65)	<0.001
3–4 days (vs. none)	5.37(4.57–6.30)	<0.001	1.80 (1.49–2.19)	<0.001
>5 days (vs. none)	10.86 (9.76–12.09)	<0.001	2.37 (2.06–2.71)	<0.001
Home SHS during the past 7 days				
1–2 days (vs. none)	1.51 (1.30–1.76)	<0.001	1.03 (0.85–1.25)	0.751
3–4 days (vs. none)	1.47 (1.22–1.77)	<0.001	0.89 (0.71–1.12)	0.316
>5 days (vs. none)	3.55 (3.24–3.88)	<0.001	1.19 (1.03–1.37)	0.016
Outside SHS during the past 7 days				
1–2 days (vs. none)	1.99 (1.65–2.40)	<0.001	1.78 (1.45–2.18)	<0.001
3–4 days (vs. none)	4.05 (3.38–4.87)	<0.001	2.60 (2.11–3.19)	<0.001
>5 days (vs. none)	11.15 (9.61–12.94)	<0.001	3.36 (2.82–4.00)	<0.001
**Community factors**				
where to receive free cigarettes offeredby tobacco companies				
Convenience store, supermarket, department store (vs. never obtained)	10.53 (7.99–13.87)	<0.001	3.94 (2.76–5.61)	<0.001
Tobacco sale (vs. never obtained)	8.80 (5.79–13.36)	<0.001	5.59 (3.26–9.59)	<0.001
Internet café (vs. never obtained)	10.53 (7.94–13.98)	<0.001	4.55 (3.15–6.58)	<0.001
KTV or MTV (vs. never obtained)	18.75 (12.88–27.31)	<0.001	4.90 (3.04–7.90)	<0.001
On the side of the road (vs. never obtained)	6.65 (4.56–9.69)	<0.001	3.38 (2.11–5.41)	<0.001
Wedding or funerals (vs. never obtained)	5.18 (4.23–6.34)	<0.001	2.32 (1.80–2.99)	<0.001
Other places (vs. never obtained)	9.43 (7.45–11.94)	<0.001	3.22 (2.39–4.34)	<0.001
Indigenous population	1.03 (1.02–1.03)	<0.001	1.01 (1.01–1.02)	<0.001
Low-income population	1.19 (1.15–1.23)	<0.001		
College/university graduate population	0.99 (0.97–1.02)	0.47		
Migrant population	1.01 (0.96–1.05)	0.836		
Temple density	0.80 (0.74–0.86)	<0.001		
Criminal cases	1.01 (0.98–1.04)	0.472		
Fertility rate under 19 years old	1.11 (1.09–1.13)	<0.001		
Divorce rate	1.20 (1.06–1.33)	0.003		
Communicable diseases	1.02 (0.99–1.05)	0.19		

^a^ CI = confidence interval; SHS = secondhand smoke.

**Table 3:** Direct, indirect, and total effect of associated factors on current cigarette smoking incidence.

	Associated Factors	Direct Effect ^a^	Indirect Effect ^a^	Total Effect ^a^
	**Positive effect**			
1	Free cigarettes offered by tobacco company (C)	0.182	0.015	0.197
2	Home SHS (E)	0.153	0.077	0.230
3	Friends smoking (E)	0.104	0.069	0.173
4	Pocket money (P)	0.080	0.000	0.008
5	Outside of home and school SHS (E)	0.061	0.078	0.139
6	School SHS (E)	0.046	0.097	0.143
7	Indigenous population (C)	0.037	0.001	0.038
8	Parents smoking (E)	0.019	0.005	0.025
	**Negative effect**			
1	Feel less comfortable at social occasions while smoking (P)	−0.236	−0.005	−0.241
2	Feel no difference whether smoking or not at social occasions (P)	−0.170	0.203	0.033
3	Female (P)	−0.068	−0.017	−0.085
4	Feel quitting is hard (P)	−0.047	−0.001	−0.048
5	School anti-smoking education and rules (E)	−0.021	0.000	−0.021
6	Feel quitting after having smoked is harmful (P)	−0.015	−0.011	−0.026

^a^ Total effect = association between associated factors and current cigarette smoking via all paths in the model; indirect effect = this association minus the direct effect of any path from associated factors to current cigarette smoking; direct effect = the total effect minus the total indirect effect. P = personal factors; E = environmental factors; C = community factors.

## Data Availability

The data used to support the findings of this study are included within the article.

## Supplementary Materials

The following supporting information can be downloaded at: https://www.mdpi.com/article/10.3390/healthcare10030534/s1. Table S1: Overall indices related to the goodness-of-fit model. Table S2: Validation analysis.
