# Supplementary material for: Influence of Personal, Environmental, and Community Factors on Cigarette Smoking in Adolescents: A Population-Based Study from Taiwan
Source: Healthcare (Basel). 2022 Mar 14;10(3):534. doi: 10.3390/healthcare10030534 (PMC8951170; doi:10.3390/healthcare10030534)
Supplement: Supplementary file 1 [file healthcare-10-00534-s001.zip › healthcare-1588402-supplementary.pdf]

Supplement Table 1 Overall indices related to the goodness-of-fit model

Model Fit Indices		Acceptable Values	Results
Absolute Fit Indices			
	SRMR	<0.05	0.0032
	RMSEA	<0.05	0.005
Incremental Fit Indices			
	TLI	>0.9	0.999
	CFI	>0.9	1.000
Parsimonious Fit Indices			
	PGFI	>0.5	0.225

SRMR: Standardized root mean square residual; RMSEA: Root mean square error of approximation; CFI: Comparative fit Index; GFI: Goodness-of-fit statistic; PGFI: parsimonious goodness-fit-index.

Supplement Table 2 Validation analysis

Associated factors	Training model	Testing g model
	Direct effect	Direct effect
**Positive effect**		
Free cigarettes offered by tobacco company (C)	0.176	0.189
Home SHS (E)	0.153	0.154
Friends smoking (E)	0.104	0.103
Pocket money (P)	0.075	0.086
Outside of home and school SHS (E)	0.054	0.069
School SHS (E)	0.047	0.046
Indigenous population (C)	0.036	0.038
Parents smoking (E)	0.027	0.011
**Negative effect**		
Feel less comfortable at social occasions while smoking (P)	-0.251	-0.219
Feel no difference whether smoking or not at social occasions (P)	-0.188	-0.149
Female (P)	-0.069	-0.066
Feel quitting is hard (P)	-0.051	-0.043
School anti-smoking education and rules (E)	-0.015	-0.027
Feel Smoke then quit is harmful (P)	-0.013	-0.017
